# Neural correlates of visual attention during risky decision evidence integration

**DOI:** 10.1016/j.neuroimage.2021.117979

**Published:** 2021-03-23

**Authors:** John R. Purcell, Andrew Jahn, Justin M. Fine, Joshua W. Brown

**Affiliations:** a Department of Psychological & Brain Sciences, Indiana University, 1101 E. 10th St., Bloomington, IN 47405, USA; b Program in Neuroscience, Indiana University, 1101 E. 10th St., Bloomington, IN 47405, USA; c Department of Psychology, University of Michigan, East Hall, 530 Church St, #1265 Ann Arbor, MI 48109, USA

## Abstract

Value-based decision-making is presumed to involve a dynamic integration process that supports assessing the potential outcomes of different choice options. Decision frameworks assume the value of a decision rests on both the desirability and risk surrounding an outcome. Previous work has highlighted neural representations of risk in the human brain, and their relation to decision choice. Key neural regions including the insula and anterior cingulate cortex (ACC) have been implicated in encoding the effects of risk on decision outcomes, including approach and avoidance. Yet, it remains unknown whether these regions are involved in the dynamic integration processes that precede and drive choice, and their relationship with ongoing attention. Here, we used concurrent fMRI and eye-tracking to discern neural activation related to visual attention preceding choice between sure-thing (i.e. safe) and risky gamble options. We found activation in both dorsal ACC (dACC) and posterior insula (PI) scaled in opposite directions with the difference in attention to risky rewards relative to risky losses. PI activation also differentiated foveations on both risky options (rewards and losses) relative to a sure-thing option. These findings point to ACC involvement in ongoing evaluation of risky but higher value options. The role of PI in risky outcomes points to a more general evaluative role in the decision-making that compares both safe and risky outcomes, irrespective of potential for gains or losses.

## 1. Introduction

Behavioral and neural studies of value-based decision-making have primarily focused on factors driving choice outcomes. Extensive neuroimaging work has shown neural correlates of different option values during decision-making, including the anterior cingulate cortex (ACC), frontal gyri, insular cortices, and striatum (Christopoulos et al., 2009; Hare et al., 2011; Kuhnen and Knutson, 2005). The process of decision-making also involves risk evaluation that decision-makers integrate into final choice options (Fiedler and Glöckner, 2012; Glickman et al., 2019), and neural sensitivity to potential gains and losses has correlated with visual attention during decision-making (Häusler et al., 2016). Neurological correlates have been thoroughly investigated using differing definitions of risk (Christopoulos et al., 2009; Fukunaga et al., 2018). However, it remains unknown if these correlates are related to the inherent attentional process of evidence integration prior to decision-making, ultimately driving choice selection.

Neuroimaging of risky choices has implicated both the dorsal ACC (dACC) and insular cortices in encoding risk (Brown and Alexander, 2017; Brown and Braver, 2007; Gehring and Willoughby, 2002; Preuschoff et al., 2008). Existing theories suggest that the ACC may driving risk avoidance (Fukunaga et al., 2012; Paulus and Frank, 2006), yet other theories posit that the ACC drives motivated, effortful behavior despite risks and costs (Holroyd and Yeung, 2012; Kolling et al., 2014; Parvizi et al., 2013). Similarly, non-human primate literature implicates the ACC in signaling predicted outcomes, prediction error, and evidence accumulation (Hayden and Platt, 2010; Isomura et al., 2003), as well as value coding of decision choices (Azab and Hayden, 2017; Kennerley et al., 2006). Hunt et al., (2018) even observed dACC neural firing signaled belief updating via visual attention, and evidence integration preceding decision choice.

Within insula, the anterior portion (AI) activation has been shown to correlate with uncertainty and risk evaluation during decision-making (Huettel et al., 2005; Mohr et al., 2010; Volz et al., 2004) and during outcome anticipation following choice (Critchley et al., 2001; Preuschoff et al., 2006; Preuschoff et al., 2008). Beyond coding an option’s uncertainty or riskiness, increased AI activity has been associated with risk-averse behavior (Huettel, 2006; Kuhnen and Knutson, 2005; Rudorf et al., 2012; Venkatraman et al., 2009). For example, insular lesions have been associated with placing higher wagers in gambling and less sensitivity to risk (Clark et al., 2008).

Although substantial evidence supports the role of ACC and insula in risky decisions, it remains an open question how they relate to the evidence integration process that precedes choice. On one hand, sequential sampling frameworks and models applied to value decisions presume that weight in favor of choosing one option or another is continuously integrated (Ratcliff and Smith, 2004). The rate of integration, and therefore reaction time, is often presumed to arise from the difference in option values. Furthermore, some of these frameworks presume an integration that continues independent of on-going attentional foveation (Glöckner and Herbold, 2011; Stewart et al., 2016). In contrast, there is substantial evidence that risky decision-making involves a comparator, selective integration process that is attributed to moment-by-moment attentional allocation (Aimone et al., 2016; Glickman et al., 2019). The connection between visual attention and integration of option values is supported by findings demonstrating a relationship between evidence accumulation rates, decisions, and visual attention (Cavanagh et al., 2014; Fisher, 2017; Glaholt and Reingold, 2011; Krajbich and Rangel, 2011; Mullett and Stewart, 2016; Thomas et al., 2017). This alternative behavioral framework raises the issue of whether brain regions linked to risky decision-making are also involved in the attentional valuation processes preceding choice.

The present study addressed whether or not the dACC and insula predict potential decisions (i.e. riskier vs. safer actions), and whether or not activation in these regions changes as a function of visual attention on choice options. The task was developed to encourage decision-making between similarly preferable sure-thing and gamble options, without any components of reinforcement, punishment, or any form of feedback learning which would impact performance between the initial and final trials. The dACC is implicated in the process of maintaining previous and potential value of choices in order to guide online decision-making behavior (Hunt et al., 2018; Seo and Lee, 2007; Wittmann et al., 2016), even representing the reward value of options ultimately not chosen (Hayden et al., 2009). These findings overall suggest that dACC may encode value related to more positively valenced choices (Kennerley et al., 2011; Wallis and Kennerley, 2011). We hypothesized ACC would be more sensitive to reward value despite risk, and specifically that neural activation within the dACC would correlate positively with foveations on the most rewarding value of the potential outcomes prior to decision-making. Specifically, the dACC is expected to be most active during visual foveation on the stimulus indicating the risky reward prospect of a gamble.

The AI has been implicated in formulating representations of potential disadvantageous outcomes or losses, as activation has been greater as uncertainty risk increases (Fukunaga et al., 2012), even when controlling for reward probability or expected value (Huettel, 2006). Additionally, AI activation preceding choice has predicted switching from risky to safer choices in uncertain risk-taking (Kuhnen and Knutson, 2005) and choices minimizing potential losses in risky gambling (Venkatraman et al., 2009). In an economic purchasing task, the AI also exhibited greater activation while viewing higher priced goods and deactivation during purchasing (Knutson et al., 2007). Consistent with its purported role in loss-aversion, the AI is expected to exhibit greater activation during visual foveation on the stimulus indicating the risky loss prospect of a gamble.

## 2. Materials & methods

### 2.1. Participants

Data was collected from 18 right-handed participants (10 female; mean age=23.44 years, SD=2.91) who were compensated $25/hour. Participants reported no history of psychiatric or neurological disorder, and no current use of psychoactive medications. Participants were trained on the task on a computer outside of the scanner until they gave verbal confirmation that they understood the task. Our sample size of 18 subjects is appropriately powered to detect an effect size of Cohen’s d=0.67, assuming a nominal alpha=0.05 and 80% power, and is consistent with other studies of concurrent eye-tracking and fMRI (e.g. Bonhage et al., 2015; Kliemann et al., 2012; Wilbertz et al., 2018).

### 2.2. Procedure

**Fig. 1.**
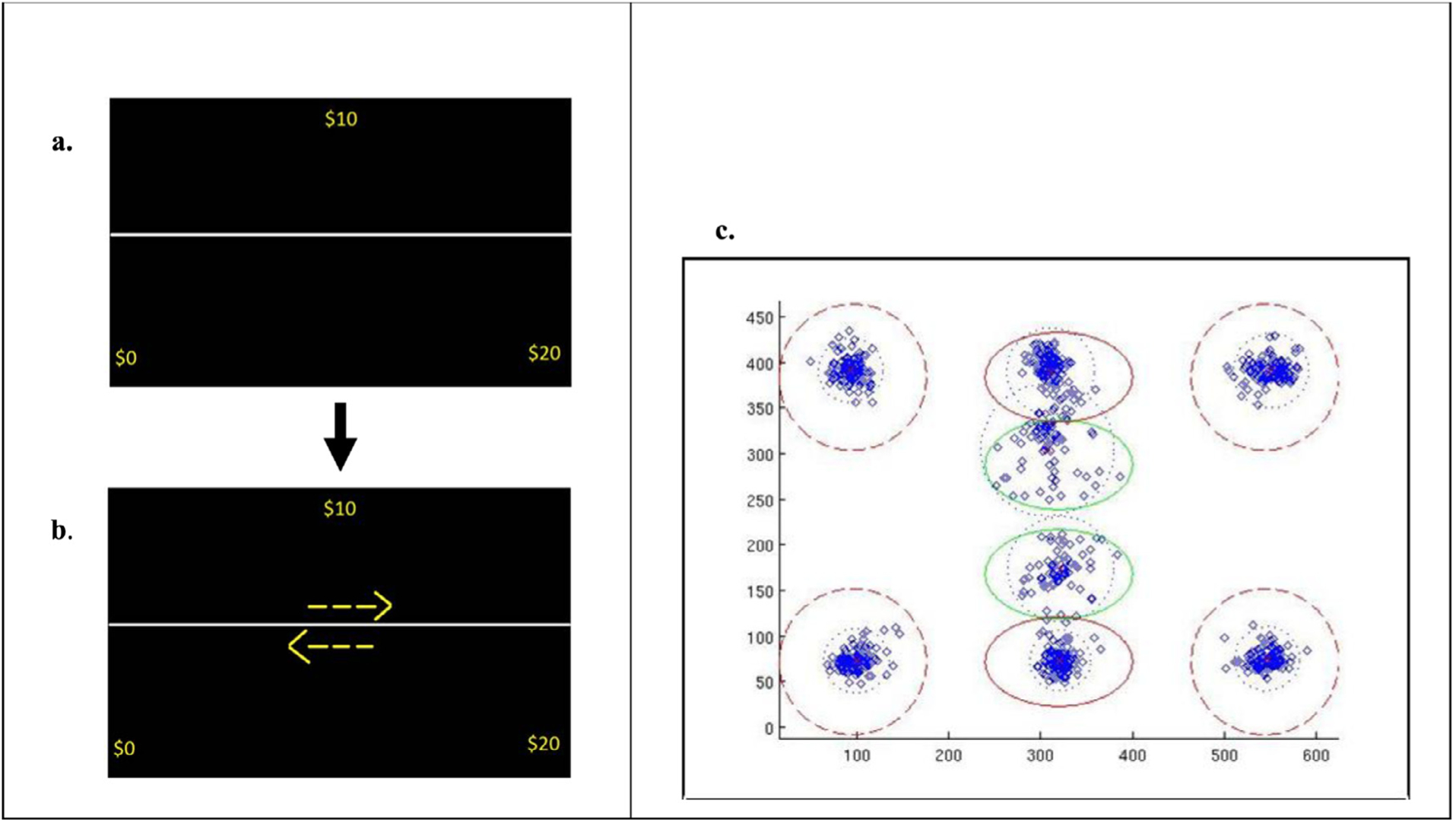
Task Trial and Example Visual Fixations. 1a: Top Right: Presentation of Gamble & Sure-Thing Amounts (7 seconds). 1b. Bottom Right: Presentation of Cue Choices. Left:1c: Visual fixations across all trials for a single subject.

During fMRI scanning, participants were presented with a choice of a sure-thing (ST) or a gamble, counterbalanced to appear on opposite halves of the screen (either top or bottom; Fig. 1a and 1b). The ST consisted of a single, positive value that would be received with a 100% probability if chosen^1^. The gamble option, on the other hand, consisted of two monetary values, one which was higher than the ST (G-Reward), and another which was either negative or null (G-Penalty). Participants were informed that there was an equal probability of receiving either gamble value (i.e. 50/50). Each pair of options was presented for 7s before cues appeared in the form of arrows, enabling participants to press a button to select either the ST (ST-Arrow) or the gamble (G-Arrow; Fig. 1b). The delayed cue enabled the cognitive deliberation processes to be isolated from the motor response. There were seven different gamble configurations, each with a consistent G-Reward value across all trials within the configuration (i.e. $10, $50, $100, $200, $500, $1000, $5000) irrespective of subject choice. Each configuration’s initial trial began with a G-Penalty of $0 and the ST worth 33% or 66% of the G-Reward (Table S1) in order to isolate neural activation associated with within-trial comparison-based value (Vlaev et al., 2011). In subsequent trails, the ST and G-Penalty values titrated as a function of whether the gamble or sure-thing was chosen on preceding trials (see supplementary materials), resulting in a narrowing the range of ST values according to the subject’s choices. The goal of the titration is to keep the probability of choosing the gamble at around 50%, so that the ST converges to the certainty equivalent. The expected value of the gamble was generally not the same as the value of the ST. Nevertheless, throughout the session, the ST values for each trial converged to the certainty equivalent of the gamble value, with an algorithm similar to that of Paulus and Frank (2006). In other words, as the session progressed, the ST and G-Loss values were adjusted in an effort to encourage subjects to choose the gamble and ST options with similar liklihood (see supplementary materials). Subjects completed as many trials out of 56 as possible within 8 minutes (M=54.83, SD= 4.22).

### 2.3. Eyetracking data collection

While inside the scanner, an eye-tracker (Eyelink 1000; SR Research Ltd., USA) recorded eye movements at a 1KHz sampling rate. The eye position at each point in time was coded as foveating on the ST, G-Reward, G-Penalty, ST-Arrow, G-Arrow, or elsewhere on the screen, yielding six regressors. Each of these six regressors was convolved with a hemodynamic response function and then sub-sampled every 2000 milliseconds for entry into a general linear model of the neuroimaging data. In order to ensure that only foveations of interest were analyzed, several steps were taken to filter the data. First, only foveations which occurred between the start of each trial and the button response from the participant were analyzed. Second, screen regions of interest (SROIs) were generated with circles and ellipses with diameters of around 80 pixels to maximize discrimination between fixations on different stimuli as described below.Fig.1 c shows a sample analysis of a participant’s foveations across trials. Dashed circles indicate locations of gambles which always appeared in corners of the screen, and only in the top or bottom for a given trial. Solid red ellipses indicate locations of the ST, which always appeared in center of screen on the top or bottom. Top and bottom locations for ST and gambles were counterbalanced. Solid green ellipses represent the locations of the arrow cues that indicate the response mappings, (i.e. which button should be pressed for the ST and which button should be pressed for the gamble). Blue diamonds represent eye foveations at each location. A red “x” indicates mean X and Y coordinate for eye foveations within each region. Blue dotted circles represent refined circles with radius of mean X,Y coordinate pair plus 2 * standard deviation of X, Y coordinate pair for that region. Only foveations falling within refined SROIs were used for analysis in the General Linear Model (GLM).

In some cases, adjacent SROIs overlapped, meaning that foveations falling in the region of overlap are ambiguous in terms of which SROI they should be assigned to. In order to resolve the ambiguity, we assign the foveation to the SROI with the nearest (normalized) center as follows. First, the following formula was applied to each coordinate pair:

$$d_{i}=\sqrt{{\left(\left(x_{i}-\text{mean}(x)\right)/\sigma x\right)}^{2}-{\left(\left(y_{i}-\text{mean}(y)\right)/\sigma y\right)}^{2}}$$

Where *d*_*¡*_ indexes each foveation. This formula takes the weighted x-and y-coordinate difference for each foveation compared to the mean center coordinate pair of each refined SROI. This creates a 1 × 8 vector for each weighted distance from each SROI. The coordinate pair is assigned to the ROI with the minimum distance in the vector. These refined foveation coordinates are then written out into a space-delimited text file and converted into timing information for the GLM.

### 2.4. fMRI acquisition and data preprocessing

The experiment was conducted with a 3 Tesla Siemens Trio scanner using a 32-channel head coil and the imaging data acquired at a 30° angle from the anterior commissure-posterior commissure line to maximize orbital and ventral sensitivity (Deichmann et al., 2003), using a gradient echo T2* weighted echo planar imaging sequence, [35 × 3.8 mm interleaved slices; TE=25 ms; TR=2000 ms; matrix, 64 × 64 voxels; field of view, 192 × 192 mm]. One run of data was collected with 360 functional scans. High resolution T1 weighted images for anatomical data were collected at the end of each session.

SPM5 (Wellcome Department of Imaging Neuroscience, London, UK; www.fil.ion.ucl.ac.uk/spm) was used for preprocessing and data analysis. The data for each participant was slice-time corrected, realigned using a 6-parameter rigid body spatial transformation, coregistered to their structural image, the structural was normalized to the standard Montreal Neurological Institute (MNI) space and the warps were applied to the functional images, and then the functional images were spatially smoothed using an 8mm Gaussian kernel. A minimum significant cluster size of 74 voxels was determined using AFNI’s 3dClustSim (nearest neighbor=1, pthr=.001, *α*=.05), using a recently compiled version to avoid problems with alpha inflation of cluster sizes associated with earlier versions (Eklund et al., 2016).

## 4. Results

### 4.1. Behavioral eyetracking analyses

Total time spent foveating each SROI was divided by the number of trials for each subject (M=53.67, SD=4.67) to account for any difference in missing trials. Paired-sample T-tests were used to investigate differences in time spent foveating each SROI.

#### 4.1.1. Average foveation duration between gamble and sure-thing chosen trials

**Fig. 2.**
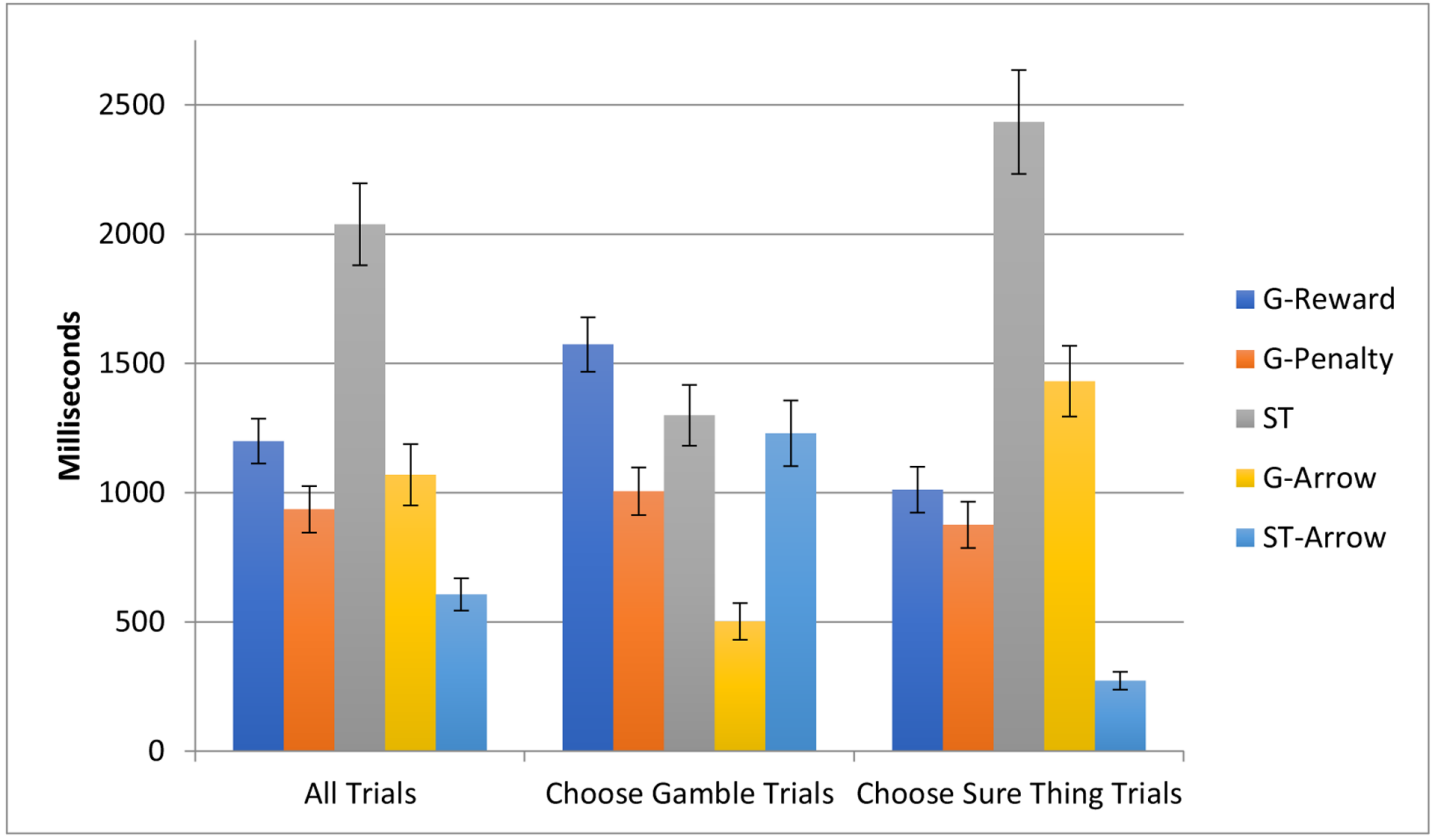
Average Foveation Duration with Standard Error Bars. Foveations Across All Trials, Trials in which the gamble was chosen, and trials in which the sure-thing was chosen. Screen Regions of Interest (SROIs): G-Reward=gamble-reward, G-Penalty=gamble-penalty, ST=sure-thing, G-Arrow=gamble arrow, ST-Arrow=sure-thing arrow.

When comparing Gamble-Chosen Trails to ST-Chosen Trials, subjects spent significantly more time foveating the G-Reward (*t*(17)=5.427, *p* < .001) and G-Penalty (*t*(17)=2.828, *p*< .012), and less time looking at the ST (*t*(17=−7.056, *p*<.001); Fig. 2). Surprisingly, subjects spent significantly more time looking at the ST-Arrow (*t*(17)=−8.174, *p*<.001) and less time looking at the G-Arrow (*t*(17)=8.679, *p*<. 001). There was no statistical difference in total time spent foveating on both arrows combined (*t*(17)=.245, *p*=.809).

#### 4.1.2. Average foveation duration within gamble chosen trials

Within the trials in which subjects chose the gamble, significantly more time was spend foveating on the G-Reward than on G-Penalty (*t*(17=5.446, *p*<.001), G-Reward than on the ST (*t*(17=5.536, *p*<.001), and the ST than the G-Penalty (*t*(17=2.536, *p*=. 01). Surprisingly, more time was spent foveating on the ST-Arrow than on the G-Arrow (*t*(17=6.193, *p*<. 001).

#### 4.1.3. Average foveation duration within sure-thing chosen trials

Similarly, within the trials in which subjects chose the ST, significantly more time was spend foveating on G-Reward than on G-Penalty (*t*(17=3.363, *p*=.002) and the ST than the G-Penalty (*t*(17=7.809, *p*< .001). However, more time was spent foveating on the ST than on the G-Reward (*t*(17=6.849, *p*<. 001). Surprisingly, more time was spend foveating on the G-Arrow than on the ST-Arrow (*t*(17=9.879, *p* <. 001).

### 4.2. fMRI analyses

#### 4.2.1. Visual attention analyses

All activation cluster sizes, locations, and significance values may be found in Table 1. BOLD activation during foveations on the gamble-reward (G-Reward), gamble-penalty (G-Penalty), and sure-thing (ST) options were first contrasted with activation during foveations within areas outside of the five SROIs within each trial (Table S2). Next differences in neural activation during foveations on each of the SROIs were examined.

**Fig. 3.**
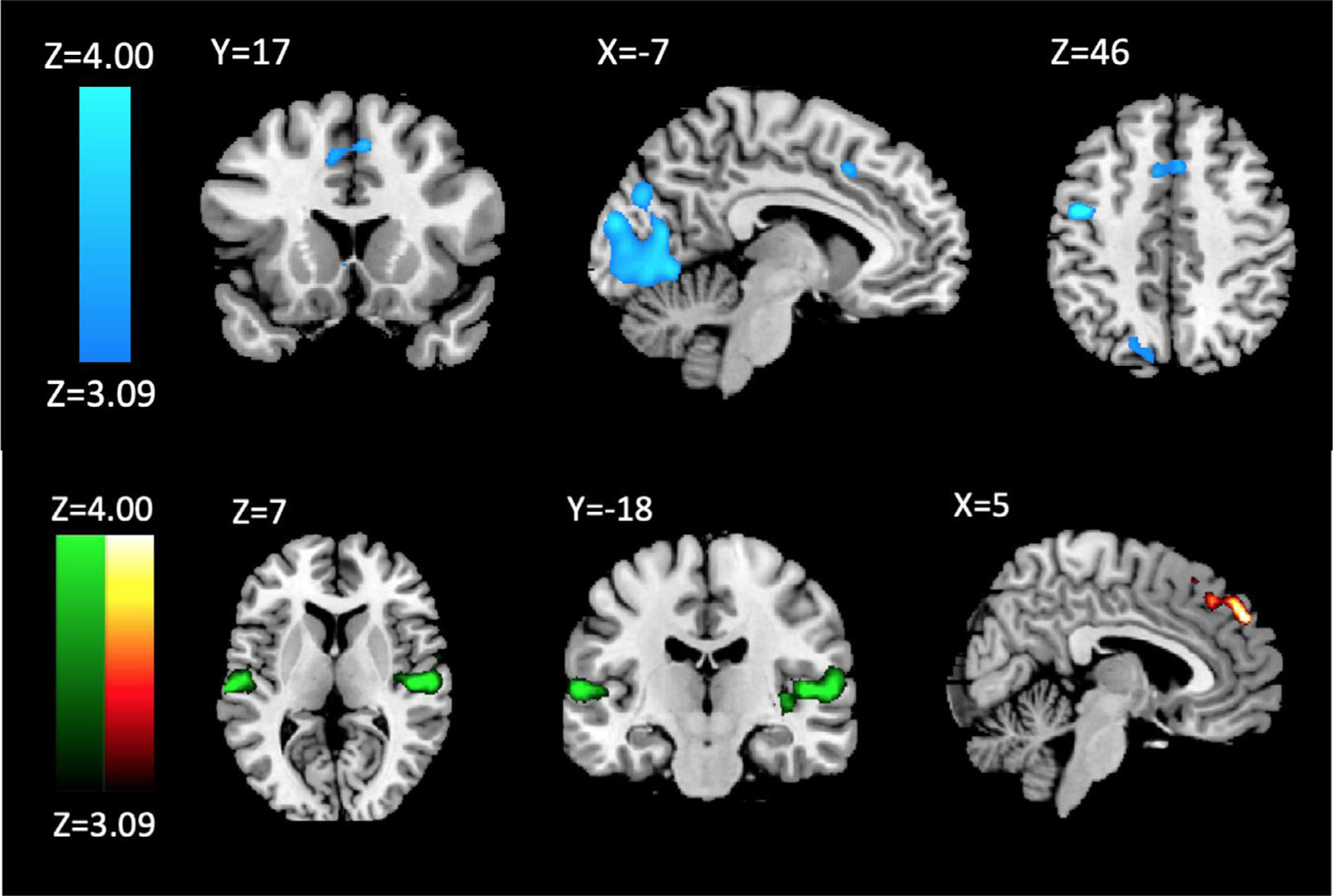
Above (Blue): Activation During GambleReward-GamblePenalty Foveations; Bottom Left (Green): Activation During GamblePenalty-GambleReward Foveations; Bottom Right (Orange): Activation during SureThing-GambleReward+GamblePenalty that correlated with amount of time foveating on (GambleReward+GamblePenalty)/SureThing.

We first asked what regions might represent evaluating evidence of reward or penalty associated with an option. Contrasting the foveations on the G-Reward option with those on the G-Penalty option revealed greater activation in the left premotor cortex (Fig. 3) and occipital lobe. A cluster within the dACC approached significance at the whole-brain level and reached significance (MNI −8,16,46; *k*=50 voxels; peak voxel z-value=3.59, *p*=0.01) via a small volume correction using WFU PickAtlas utilizing a mask of Brodmann’s area 24 and 32 with 0<Y<36 and Z>5, with a dilation of 3mm. These findings are consistent with increased activation within the ACC and central sulcus associated with integrating reward or salience of stimuli prior to risky decision-making (Christopoulos et al., 2009; Krain et al., 2006; Litt et al., 2011).

Next, we looked for regions that might integrate aversive cue information. To do this, we tested the contrast of foveations on G-Penalty minus G-Reward. This contrast revealed bilateral pattern of activity in the posterior insulae (PI). Though counter to our hypothesized anterior insula activation, these findings are consistent with the posterior insula’s purported role in risk avoidance, assessing aversive outcomes, and salience signals associated with attention, arousal, and motivation (Canessa et al., 2013; Litt et al., 2011).

We then compared activation during foveations between gamble outcomes and the ST in order to investigate differences attributable to risky or safe evidence integration. Less activation in the PI and putamen was found during foveations on the ST relative to G-Reward, G-Penalty, and a combination of G-Reward and G-Penalty. These findings may reflect the ST option’s lack of risk, as activation within these regions has been associated with risky compared to safe choices and anticipating outcomes following risky choices with greater loss amounts (Canessa et al., 2013; Paulus et al., 2003).

#### 4.2.2. fMRI correlational analyses

To investigate potential associations between behavior and activation, we correlated neural activation during foveations of the possible outcomes by percent of gambles chosen across trials. At the whole-brain level, no significant clusters emerged, but during foveation of G-Reward – G-Penalty activation within the aforementioned small-volume dACC mask correlated with the percent of gambles chosen (MNI −2,24,28; *k*=28 voxels; peak voxel z-value=3.54, *p*=.03, cluster corrected within small volume region). Taken together, activation within this small region of the dACC may be consistent with accounts of integrating information in favor of approach behavior despite risk (Kolling et al., 2014), though these results are interpreted with caution considering the small cluster of activation and marginal significance.

**Fig. 4.**
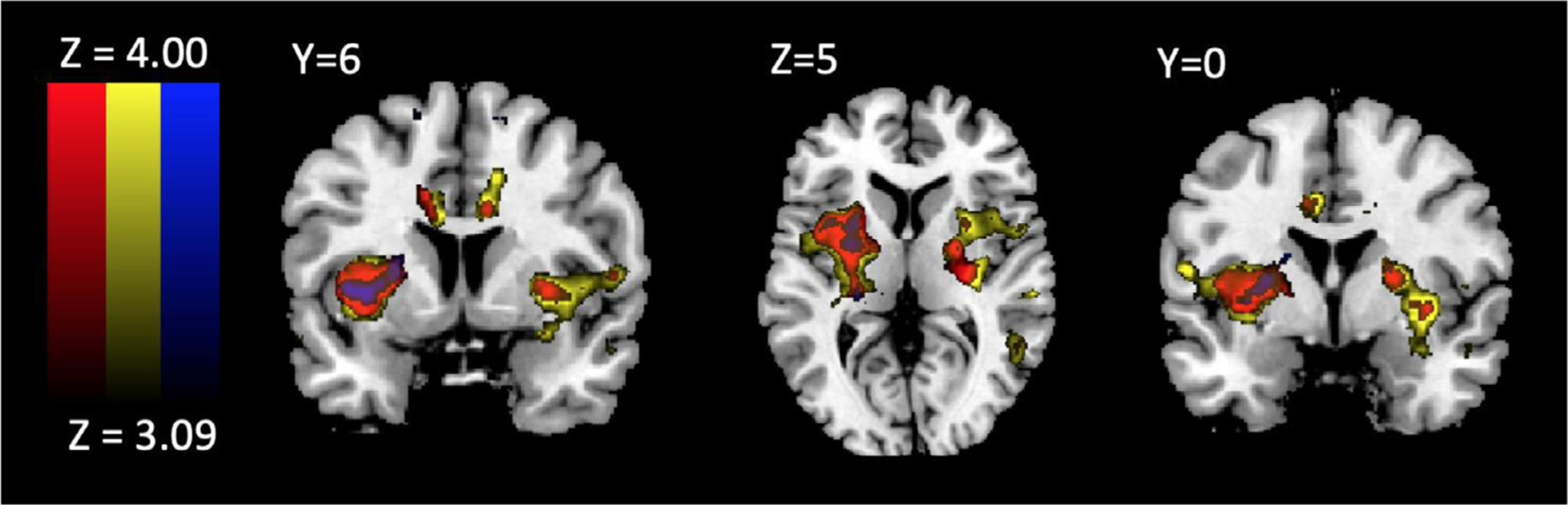
Activation During GambleReward+GamblePenalty-SureThing (Red), GamblePenalty-SureThing (Yellow), GambleReward-SureThing (Blue) foveations.

To further identify neural regions associated with visual attention, correlations between time spent foveating to the SROIs and neural activation were investigated. Activation in the frontal cortex during foveations on the ST compared to gamble options (G-Reward + G-Penalty) was correlated with more overall foveations on the gamble options relative to the ST (Fig. 3). This may be a kind of infrequency effect, as the less time subjects spent looking at the ST option, the more activation occurred when they did look at the ST. Activation within this region was also greater when comparing foveations on the ST to general, non-SROI foveations (Fig. 4).

### 4.3. Exploratory analyses

Neural activation based upon subsequent decision-choice was also investigated, with results lacking strong convergence across contrasts (Table S2). However, activation in the medial PFC may correspond more with evidence accumulation that aligns with the decision to be made. This is evident in the G-Penalty foveations relative to non-SROI saccades during ST-Chosen trials (MNI 10,−6,56; *k*=105 voxels; peak voxel z-value=3.92, *p*=0.02), G-Reward foveations in Gamble-Chosen relative to ST-Chosen trials (MNI 8,−12,54; *k*=315 voxels; peak voxel z-value=4.43, *p*<0.001), and G-Penalty relative to G-Reward foveations on ST-Chosen trials (MNI 2,−12,50; *k*=2755 voxels; peak voxel z-value=4.29, *p*<0.001). Notably, while bilateral anterior insulae clusters emerged during G-Penalty foveations regardless of choice, this effect was likely driven by ST-Chosen Trials (Left: MNI −42,20,0; *k*=89 voxels; peak voxel z-value=3.66, *p*=0.036; Right: MNI 48,28,−2; fc=497 voxels; peak voxel z-value=4.76, *p*<0.001), as these clusters did not emerge in Gamble-Chosen Trials. The results of these exploratory analyses are interpreted with caution as our task was not designed in a fashion that allowed for equal sampling between ST-Chosen Trials and Gamble-Chosen Trials.

## 5. Discussion

The anterior cingulate cortex (ACC) and insula have been implicated in encoding the effects of risk and value on decision outcomes. We investigated the evidence integration processes that precedes choice in order to determine how activation within these regions is related to attention. Concurrent fMRI and eye-tracking were used to investigate the roles of the ACC and insula in the evidence integration process preceding choice selection in risky decision-making. These findings complement substantial literature implicating both regions in risk and value processing at the time of choice and outcome evaluation. In particular, they implicate the ACC in integrating the value for pursuing risky decisions despite risk and the posterior insula in integrating risky choices.

Behaviorally, our eye tracking results were consistent with literature suggesting that gaze duration can index decision choice (e.g. Cavanagh et al., 2014; Sheng et al., 2020). When choosing the gamble, subjects foveated each of the G-Reward and G-Penalty more, and the ST less. This suggests an attentional bias consistent with choice. Our design of separating the G-Penalty and G-Reward from the ST option, allowed for the individual parsing of these gamble parameters. However, most research in decision-making using eye-tracking has presented gambles in the form of a two-armed bandit (with four stimuli for each gamble outcome magnitude and probability). Thus, the current experiment’s design allowed us to determine how risky rewards and risky losses individually enter into the attentional processes, relative to a sure-thing, to support decision-making (Stewart et al., 2003).

At the level of brain activation, our main expectations were that ACC would exhibit a prominent role in encoding attentional processes for evaluating the gain of a risky gamble. This was supported by the finding that greater ACC activation was found during foveations on the G-Reward compared to the G-Penalty option. These findings suggest dACC underlies a comparative decision process that evaluates the available gain relative to loss within a single risky choice option (Brown and Braver, 2007, 2008; Parvizi et al., 2013; Walton et al., 2007). These findings implicate the dACC in integrating evidence for reward approach despite risk. Our correlational findings provide some support for this interpretation, as increased ACC activation during G-Penalty relative to G-Reward was linked to an increased propensity for choosing the gamble option.

Beyond the ACC, the insular cortex has regularly been found to activate during risky and uncertain decision making. In particular, the Anterior insula (AI) has been shown to increase in activation with increased decision uncertainty (Huettel et al., 2005; Preuschoff et al., 2006; Volz et al., 2004). AI is also linked to real-time updating of risk, prediction error, and influencing future choice behavior (Bossaerts, 2010; Kuhnen and Knutson, 2005; Mohr et al., 2010; Preuschoff et al., 2008). Accordingly, we asked whether the AI is also involved in the evidence integration process preceding risky decision-making. While we found a relation between foveations on risky outcomes and insular activity, the centroid of this cluster was posterior rather than anterior. A possible reason for the lack of AI effect is our design differs from standard risk-taking studies. Our task did not allow for the formulation of prediction error, as subjects were told outcome probabilities (i.e. 50/50) but never observed the outcomes of their decisions. This difference in task parameters may relate to the lack of AI activation which has exhibited during error processing and feedback presentation (for review see Chang et al., 2013).

The finding of PI activation is not entirely surprising, and accords with previous work linking this region with integrating loss information preceding decision-making (Furl and Averbeck, 2011). Others have found PI relates to the prediction of potential losses or magnitude of impending loss (Canessa et al., 2013; Paulus et al., 2003). These studies have found PI activity increases at both the point of decision choice and during the process leading up to it. Additionally, the PI has been associated with more abstract constructs related to our task such as decision salience (i.e. attention/motivation), risky “urges”, the anticipatory feeling of aversive outcomes, and even the passage of time (Droutman et al., 2015; Litt et al., 2011; Loewenstein et al., 2001; Straube and Miltner, 2011; Wittmann et al., 2010). Thus, future task designs should aim to further parse the cognitive and affective contributions of the PI in evidence integration preceding risky decision-making.

AI activity was not totally absent from our findings. Our exploratory analyses revealed bilateral AI activation clusters during foveations on the G-Penalty relative to saccades falling outside any screen region of interest (SROIs). This effect was specific to trials in which individual chose the ST. A possible interpretation is that AI is specifically involved in evaluating the most aversive, risky potential loss. These findings coalesce with previous literature implicating the AI in translating risk-aversion neural signals into risk-averse behavior (Huettel, 2006; Kuhnen and Knutson, 2005; Rudorf et al., 2012; Venkatraman et al., 2009).

Differential activation was found in brain regions broadly associated with visual processing. Preceding choice, increased activation in BA6 and BA8 while foveating reward relative to penalties may include frontal and supplementary eye fields (FEF; SEF) and premotor cortex. Our findings complement those in non-human primates that such regions are implicated in value encoding, prospective reward, performance monitoring, and choice confidence during visual search-dependent decision-making (Abzug and Sommer, 2018; Mullett and Stewart, 2016; Purcell et al., 2012; Roesch and Olson, 2003; So and Stuphorn, 2016). We also found occipital activation during G-Reward and ST foveations relative to G-Penalty. Previous findings have implicated large portions of the occipital lobe in risky decision-making, particularly during risky choice, and gain outcomes relative to loss outcomes (Engelmann and Tamir, 2009; Krain et al., 2006; Matthews et al., 2004). While occipital activation has been ubiquitous in some risky decision-making studies (Huettel, 2006; Korucuoglu et al., 2020), others suggest greater activation during decisions for both high risk and high reward (e.g. Ernst et al., 2004; Rao et al., 2008). Our findings indicate that while occipital activation was greater for all gamble stimuli relative to non-stimuli foveations (Table S2), there was greater activation for rewarding outcomes relative to penalties (i.e. G-Reward and ST foveations relative to G-Penalty).

In conclusion, our finding that ACC was sensitive to differences in risk agrees with a large literature pointing to its role in value search and control over decision processes (Kolling et al., 2016). However, others and our own previous findings have found that risky decision processing involves both ACC and AI, rather than PI (Fukunaga et al., 2012; Xue et al., 2010). This opens the question of whether differing regions of insular cortex are involved in differing components of decision-making such at attention allocation or value integration. Some intuition may be gleamed from structural connectivity studies, as the PI has increased connectivity to salience regions, rather than those implicated in value processing. For example, non-human primate studies have demonstrated that, compared to the AI, PI regions have stronger structural connections to cingulate (and parietal cortex; Mesulam and Mufson, 1982). Similar support comes from diffusion-weighted imaging in humans (Cloutman et al., 2012; Jakab et al., 2012).

## 6. Limitations

There are several limitations to consider with this research. The first is that the duration of looking time imposed upon participants before decision-making may make the behavioral results less comparable to previous research as the majority of previous literature focuses on speeded decision-making or decision-making without an imposed waiting period. Second, despite our intentions to control the choice probabilities via titrating gamble and ST amounts, subjects varied in the number of risky gambles chosen (supplementary material). Previous research has observed differing fixation patterns related to risky decision-making (e.g. subjects who choose the risky gamble pay less attention to risky loss amounts; Brandstätter and Körner, 2014), thus future studies would be improved by more rigorous methods of ensuring within-subject parity between the number of risky and not-risky options chosen in order to assure neural activation was not overly impacted by higher or lower number of within-subject trials of risk-taking behavior. Third, while titrating gambles provides further specificity of within-subject risk-taking, it constrains the overall range of risky decisions between subjects. This method does not allow for as objective of comparisons across subjects, as the ‘risk environment’ changes as a function of subject choice. Therefore, replicating our findings with a uniform risk environment across subjects may paint a more complete picture of the neural correlates of risky decision-making. Fourth, while subjects were instructed to evaluate risk according to the values and outcome probabilities of the gambles within each trial, it is possible that the lack of outcome feedback and lack of monetary incentivization rendered these amounts inconsequential (Barron and Erev, 2003; Krain et al., 2006). Additionally, differences in risk-taking behavior and brain activity related to task performance bonus payment have been reported (Schmidt et al., 2019). Future research would likely benefit from incentivizing task performance via monetary bonuses. Fifth, the lack of activation differences in several brain regions was surprising. Most notably, OFC cell firing in non-human primates and ventromedial prefrontal cortex (VMPFC) neural activation in humans has been associated with visual fixations on valuable cues (Häusler et al., 2016; Lim et al., 2011; McGinty et al., 2016; Xie et al., 2018). Thus, the dearth of activation difference in the OFC or VMPFC when comparing G-Reward and G-Penalty foveations is surprising especially considering findings in past work with similar scan parameters and analysis methods (Fukunaga et al., 2012). Despite preemptively collecting data at a tilt ~30° from axial, signal dropout and image distortion remain pernicious issues when examining OFC activation, due to its close proximity to air-filled sinuses which cause magnetic field distortions in the vicinity (Deichmann et al., 2003).

Finally, computational modeling approaches may be utilized to evaluate dynamic visual attention factors contributing to choice behavior. This was not feasible in the current experimental design due to the imposed looking time and small number of trials. Still, such modeling approaches in a future design might include drift diffusion, parallel constraint satisfaction, heuristic and decision-making theories (Glöckner and Betsch, 2008; Glöckner and Herbold, 2011; Krajbich and Rangel, 2011; Su et al., 2013). While these models have aided in understanding choice behavior across contexts and experimental designs, and overarching theme is that the most visually attended-to option is typically chosen, independent of option probabilities and magnitudes (Orquin and Loose, 2013; Stewart et al., 2016). Future experimental designs might build from this computational literature in order to understand neural processes that contribute to the dynamic, within-trial process of evidence accumulation (e.g. Lim et al., 2011).

## Supplementary Material

supplement

## Figures and Tables

**Table 1:** Primary Neuroimaging Results.

Region	Laterality	Cluster Size	Peak X	Peak Y	Peak Z	Max stat Z	P Cluster Corrected
**GambleReward − GamblePenalty**							
Occipital Lobe	Bilateral	5612	16	−84	2	4.71	< .001
Premotor Cortex(BA 6)	Left	239	−44	−4	44	4.97	< .001
Anterior Cingulate(BA 8)	Bilateral	50†	−8	16	46	3.59	< .01*
**GamblePenalty − GambleReward**							
Posterior Insula(BA 13)	Right	693	48	−18	6	4.62	*<* .001
Posterior Insula(BA 13)	Left	248	−62	−20	6	4.49	*<* .001
**GambleReward − SureThing**							
Putamen/ PosteriorInsula (BA 49/13)	Left	323	−34	6	0	4.46	*<* .001
Thalamus (BA 50)	Right	167	22	−20	12	4.22	=.005
Cerebellar Lobule VI	Right	142	12	−70	−12	4.21	= .012
**SureThing − GambleReward**							
None	none	none	none	none	none	none	none
**SureThing − GamblePenalty**							
Parietal (BA 39)	Right	483	34	−58	44	4.59	<.001
Occipital (BA 18)	Right	325	36	−84	−8	4.48	<.001
Occipital (BA 17)	Bilateral	1681	12	−72	12	4.27	<.001
Parietal (BA 40)	Right	113	48	−40	48	3.85	=.034
Anterior Cingulate (BA 8)	Bilateral	55†	6	20	40	3.58	=.007*
**GamblePenalty − SureThing**							
Putamen/ Posterior Insula (BA 49/13)	Left	2413	−26	8	2	6.27	<.001
Putamen/Posterior Insula (BA 49/13)	Right	3856	26	−4	8	6.19	<.001
Cingulate Gyrus(BA 32)	Right	205	10	8	32	5.66	<.001
Inferior Parietal Lobe (BA 40)	Left	3333	−50	−26	20	5.19	<.001
Postcentral Gyrus (BA 1)	Right	808	30	−38	52	4.91	<.001
CerebellarLobule V	Right	293	16	−52	−22	4.78	<.001
Middle Frontal White Matter(BA 9)	Left	129	−24	36	20	3.95	=.19
Cerebellar Crus II	Bilateral	240	−16	−74	−38	3.87	=.001
Precentral Gyrus (BA 6)	Right	116	24	−14	58	3.81	=.030
**GambleReward + GamblePenalty − Surething**							
Superior Parietal Lobe (BA3/4)	Left	1568	−38	−34	54	5.97	*<* .001
Putamen (BA 49)	Right	1009	26	−4	8	5.86	*<* .001
Putamen/Posterior Insula (BA 49/13)	Left	1486	−30	8	2	5.83	*<* .001
Cingulate Cortex (BA 32)	Right	109	10	10	30	4.60	= .002
Cingulate Cortex (BA 32)	Left	158	−10	14	30	4.45	*<* .001
Inferior Parietal (BA 22)	Right	482	50	−36	20	4.21	*<* .001
Cerebellar Lobules V/IV	Right	119	28	−44	−30	4.19	= .025
Middle Temporal Gyrus (BA 21)	Right	115	50	−36	−6	4.12	= .030
Precuneous Cortex (BA 7)	Right	232	10	−52	56	4.09	= .001
**SureThing − GambleReward + GamblePenalty**							
Occipital (BA19)	Right	266	38	−82	−10	4.35	*<* .001
Parietal (BA 39)	Right	276	36	−54	40	4.24	*<* .001
Lateral Prefrontal Cortex (BA 9)	Right	125	40	30	20	4.02	= .020
Parietal (BA 40)	Right	109	48	−40	48	3.85	= .037
Occipital (BA 17)	Bilateral	152	8	−70	14	3.60	= .008
**Correlation Findings**							
Region	Laterality	Cluster Size	Peak X	Peak Y	Peak Z	Max stat Z	P Cluster Corrected
**PercentGambleChosen and G-Reward − G-Penalty (negative loading)**							
Anterior Cingulate (BA 32)	Bilateral	28†	−2	24	28	3.54	=.03*
**(G-Reward + G-PenaltyFixation)/STFixation and ST − G-Reward + G-Penalty**							
Superior Frontal Gyrus (BA 9)	Right	138	4	50	36	4.46	=.011
**(G-Reward + G-PenaltyFixation)/STFixation and ST − Saccade**							
Superior Frontal Gyrus (BA 9)	Bilateral	152	6	50	36	4.33	=.013

BA = Brodmann Area.

† = A minimum significant cluster size of 74 voxels at a whole brain level was determined using AFNI’s 3dClustSim (nearest neighbor=1, pthr=. 001, *α*=. 05).

* = Small volume corrected using WFU PickAtlas Utilizing a mask of Brodmann’s area 24 and 32 (−2, 24, 28) with 0 < Y < 36 and Z > 5, with a dilation of 3mm. Correlations reported compare duration of percent of gamble chosen and duration of foveations by brain activation during foveations in the screen regions of interest (SROI)s during the task.

G-Reward=Gamble Reward, G-Penalty=Gamble Penalty, ST=Sure-Thing.

## Footnotes

1 One extremely risk-averse subject was given a ST value of 0 across six trials.
