# Supplementary material for: Neural correlates of visual attention during risky decision evidence integration
Source: Neuroimage. Author manuscript; Available in PMC 2021 Jul 1. (PMC8159858; doi:10.1016/j.neuroimage.2021.117979)
Supplement: supplement [file NIHMS1690158-supplement-supplement.docx]

Title: Neural correlates of visual attention during risky decision evidence integration

Authors: Purcell, Jahn, Fine, & Brown

**Table S1. Starting Gamble & Sure-Thing Value Magnitudes for All Trials:**

| Gamble Reward ($) | Gamble Penalty ($) | Sure-Thing ($) | Sure-Thing as fraction of Reward |
| --- | --- | --- | --- |
| 10 | 0 | 3 | 0.3 |
| 50 | 0 | 33 | 0.66 |
| 100 | 0 | 33 | 0.33 |
| 200 | 0 | 133 | 0.66 |
| 500 | 0 | 166 | 0.33 |
| 1000 | 0 | 667 | 0.66 |
| 5000 | 0 | 1665 | 0.33 |

**Cert. Equiv Task Formulas**

- Alt = Alternating value equal to either 0.333 (i.e. 1/3) or 0.667 (i.e. 2/3).
- The UpperBound and LowerBound are values that are specific to each of the seven gambles individually and change depending on choice.
- The GambleReward amount is static across all trials.
- n = trial number for a given gamble

Initial UpperBound = GambleReward

Initial LowerBound = GamblePenalty

**If SureThing is Chosen on trial n-1:**

LowerBound _n_  = LowerBound_n-1_

UpperBound _n_ = SureThing_n-1_

GamblePenalty _n_ = GamblePenalty_n-1_

**If Gamble is Chosen on trial n-1:**

LowerBound _n_ = SureThing_n-1_ – (0.05(UpperBound _n-1_ – SureThing_n-1_))

UpperBound _n_ = UpperBound_n-1_

GamblePenalty _n_ = GamblePenalty_n-1_ – 0.05(GambleReward - GamblePenalty _n-1_)

**SureThing values are computed as:**

SureThing _n_ = Alt * (UpperBound _n-1_-LowerBound _n-1_) + LowerBound _n-1_

SureThing values were always a positive integer, (except one risk-averse subject was given six trials with a SureThing of 0), gamble-win values were always a positive integer, and gamble-penalty values were a negative integer or zero

**Table S2. Secondary Imaging Results:**

A minimum significant cluster size of 74 voxels was determined using AFNI’s 3dClustSim (nearest neighbor=1, pthr=.001, α=.05). Findings below this threshold are indicated with †

| Region  (Brodmann Area) | Laterality | Cluster Size | Peak X | Peak Y | Peak Z | Max stat Z | P Cluster Corrected |
| --- | --- | --- | --- | --- | --- | --- | --- |
| AllTrials_GamblePenalty-Saccade | | | | | | | |
| Middle Frontal Gyrus (9) | Right | 119 | 30 | 28 | 20 | 5.24 | =.017 |
| Anterior Insula (13) | Right | 587 | 36 | 14 | 0 | 5.11 | <.001 |
| Occipital Lobe (18) | Right | 409 | 36 | -86 | -8 | 5.08 | <.001 |
| Occipital Lobe (18) | Left | 415 | -32 | -92 | -12 | 4.80 | <.001 |
| Parietal Lobe (7) | Left | 155 | -34 | -44 | 42 | 4.33 | =.004 |
| Parietal Lobe (7) | Right | 517 | 30 | -64 | 56 | 4.17 | <.001 |
| Occipital Lobe (19) | Right | 187 | 42 | -66 | -12 | 3.91 | =.001 |
| Anterior Insula (45) | Left | 325 | -42 | 18 | 4 | 3.90 | <.001 |
| Cingulate Gyrus (6) | Bilateral | 121 | 10 | 16 | 50 | 3.88 | =.016 |
| Middle Frontal Gyrus (6) | Left | 197 | -30 | -2 | 52 | 3.62 | =.001 |
| AllTrials_GambleReward-Saccade | | | | | | | |
| Cingulate Gyrus (8) | Bilateral | 1469 | 8 | 14 | 48 | 5.13 | <.001 |
| Occipital Lobe (18) | Right | 675 | 34 | -84 | -8 | 4.83 | <.001 |
| Occipital-temporal Lobe (37) | Left | 806 | -42 | -52 | -18 | 4.82 | <.001 |
| Occipital Parietal Lobe (7) | Left | 1012 | -14 | -68 | 52 | 4.75 | <.001 |
| Anterior Insula (13) | Right | 138 | 34 | 24 | -2 | 4.27 | =.025 |
| Occipital Parietal Lobe (39) | Right | 299 | 32 | -70 | 28 | 3.89 | <.001 |
| Superior Frontal Gyrus (6) | Right | 120 | 28 | -6 | 50 | 3.80 | = .045 |
| AllTrials_SureThing-Saccade | | | | | | | |
| Inferior Occipital Lobe (18) | Right | 914 | 36 | -86 | -8 | 6.35 | <.001 |
| Inferior Occipital Lobe (18) | Left | 762 | -32 | -90 | -12 | 5.16 | <.001 |
| Parietal Lobe (39) | Bilateral | 1798 | 34 | -56 | 42 | 5.09 | <.001 |
| Cingulate Gyrus (8) | Bilateral | 521 | 4 | 20 | 44 | 4.91 | <.001 |
| Occipital Lobe (39) | Left | 311 | -24 | -68 | 40 | 4.06 | <.001 |
| Middle Frontal Gyrus (46) | Right | 144 | 44 | 34 | 18 | 3.95 | =.020 |
| Middle Frontal Gyrus (6) | Left | 204 | -44 | 6 | 32 | 3.94 | =.003 |

**Table S3. Exploratory Between Choose Gamble vs. Choose Surething Results:**

| Region  (Brodmann Area) | Laterality | Cluster Size | Peak X | Peak Y | Peak Z | Max stat Z | P Cluster Corrected |
| --- | --- | --- | --- | --- | --- | --- | --- |
| GambleReward_ChooseGamble-ChooseSureThing | | | | | | | |
| Posterior Cingulate Cortex (6) | Bilateral | 315 | 8 | -12 | 54 | 4.43 | < .001 |
| Postcentral Gyrus (4) | Left | 135 | -24 | -28 | 62 | 4.20 | .002 |
| GambleReward_ChooseSureThing-ChooseGamble | | | | | | | |
| none | none | none | none | none | none | none | none |
| GamblePenalty_ChooseSureThing-ChooseGamble | | | | | | | |
| Medial Temporal Gyrus (21) | Right | 105 | 66 | -36 | -2 | 5.35 | .008 |
| GamblePenalty_ChooseGamble-ChooseSureThing | | | | | | | |
| Occipital Lobe (18) | Bilateral | 317 | 8 | -92 | 20 | 4.83 | <.001 |
| SureThing_ChooseSureThing-ChooseGamble | | | | | | | |
| none | none | none | none | none | none | none | none |
| SureThing_ChooseGamble-ChooseSureThing | | | | | | | |
| none | none | none | none | none | none | none | none |
| GamblePenalty-GambleReward_ChooseSureThing-ChooseGamble (positive loading) | | | | | | | |
| Putamen (49) | Right | 86 | 32 | -10 | 0 | 4.40 | .023 |
| Postcentral Gyrus (1) | Left | 153 | -26 | -34 | 62 | 4.17 | <.001 |
| Superior Parietal Lobule (7) | Right | 80 | 20 | -44 | 62 | 3.75 | .033 |
| GamblePenalty-GambleReward_ChooseSureThing-ChooseGamble (negative loading) | | | | | | | |
| IntraCalcarine Cortex (18) | Right/Bilateral | 1013 | 8 | -72 | 4 | 4.50 | < .001 |
| Dorsal Anterior Cingulate Cortex (8) | Bilateral | 25† | 2 | 16 | 42 | 3.69 | =.022* |
| SureThing-GamblePenalty+GambleReward_ChooseGamble | | | | | | | |
| none | none | none | none | none | none | none | none |
| GamblePenalty+GambleReward-SureThing_ChooseGamble | | | | | | | |
| none | none | none | none | none | none | none | none |

* = Small volume corrected using WFU PickAtlas Utilizing a mask of brodmann’s area 24 and 32 with 0 < Y < 36 and Z > 5, with a dilation of 3mm.

**Table S5. Within Choose Sure-Thing Results**:

| Region | Laterality | Cluster Size | Peak X | Peak Y | Peak Z | Max stat Z | P Cluster Corrected |
| --- | --- | --- | --- | --- | --- | --- | --- |
| GamblePenalty-Saccade_ChooseSureThing | | | | | | | |
| Inferior Occipital Lobe (18) | Right | 347 | 36 | -88 | -10 | 4.96 | <.001 |
| Inferior Occipital Lobe (18) | Left | 331 | -32 | -94 | -12 | 4.87 | <.001 |
| Anterior Insula (47) | Right | 497 | 48 | 28 | -2 | 4.76 | <.001 |
| Parietal Lobe (40) | Right | 153 | 50 | -46 | 40 | 3.95 | =.003 |
| Supplementary Motor Area (6) | Right | 105 | 10 | -6 | 56 | 3.92 | =.021 |
| Superior Frontal Sulcus (6) | Left | 246 | -30 | -8 | 52 | 3.71 | <.001 |
| Anterior Insula (13) | Left | 93 | -42 | 20 | 0 | 3.66 | =.036 |
| Supramarginal Gyrus (40) | Left | 89 | -50 | -36 | 34 | 3.66 | =.043 |
| GambleReward-Saccade_ChooseSureThing | | | | | | | |
| Occipital Lobe (18) | Left | 911 | -26 | -88 | -12 | 5.17 | <.001 |
| Cingulate Gyrus (32/8) | Bilateral | 479 | 8 | 14 | 46 | 4.85 | <.001 |
| Inferior Occipital Lobe (19) | Right | 373 | 38 | -84 | -10 | 4.54 | =.010 |
| Parietal Lobe (7) | Right | 138 | 30 | -68 | 32 | 4.29 | <.001 |
| Occipital/Parietal Lobe (19) | Left | 556 | -30 | -78 | 22 | 4.12 | <.001 |
| Parietal Lobe (7) | Right | 98 | 14 | -72 | 42 | 3.84 | =.048 |
| SureThing-Saccade_ChooseSureThing | | | | | | | |
| Inferior Occipital Lobe (18) | Right | 803 | 36 | -86 | -8 | 5.70 | <.001 |
| Parietal Lobe (39) | Right | 1636 | 34 | -60 | 46 | 5.28 | <.001 |
| Inferior Occipital Lobe (18) | Left | 463 | -32 | -90 | -12 | 5.09 | <.001 |
| Dorsal Anterior Cingulate Cortex (8) | Bilateral | 94 | 4 | 20 | 40 | 3.88 | =.001* |
| Frontal Lobe (44) | Left | 152 | -42 | 2 | 26 | 3.69 | =.01 |
| GambleReward+GamblePenalty-Saccade_ChooseSureThing | | | | | | | |
| Occipital Lobe | Left | 946 | -28 | -90 | -14 | 5.28 | <.001 |
| Occipital Lobe | Right | 435 | 36 | -86 | -10 | 5.07 | <.001 |
| Inferior Temporal Gyrus | Right | 222 | 48 | -58 | -16 | 4.81 | =.001 |
| Superior frontal gyrus | Bilateral | 1220 | -18 | 4 | 62 | 4.74 | <.001 |
| Occipital Lobe | Right | 206 | 30 | -66 | 32 | 4.61 | =.001 |
| Occipital Lobe | Left | 671 | -12 | -72 | 54 | 4.42 | <.001 |
| GambleReward-GamblePenalty_ChooseSureThing | | | | | | | |
| Intracalcarine Cortex (17) | Bilateral | 7443 | 0 | -70 | 10 | 5.15 | < .001 |
| Cingulate Gyrus (8/32) | Bilateral | 280 | 4 | 20 | 46 | 5.08 | < .001 |
| Inferior Temporal Gyrus (37) | Left | 155 | -30 | -50 | -20 | 4.14 | = .004 |
| GamblePenalty-GambleReward_ChooseSureThing | | | | | | | |
| Posterior Insula (41) | Right | 845 | 52 | -16 | 8 | 4.56 | <.001 |
| Posterior Cingulate (24) | Bilateral | 275 | 2 | -12 | 50 | 4.29 | <.001 |
| Posterior Insula (13) | Left | 142 | -26 | -18 | 8 | 4.13 | =.006 |
| Posterior Insula (1) | Left | 237 | -56 | -18 | 10 | 4.10 | <.001 |
| Cingulate Gyrus (24/32) | Bilateral | 66† | 4 | 0 | 38 | 4.01 | =.007* |
| Postcentral Gyrus (4) | Right | 132 | 22 | -26 | 50 | 3.89 | =.009 |
| Posterior Insula (6) | Right | 101 | 58 | 2 | 2 | 3.88 | =0.033 |
| GambleReward+GamblePenalty-SureThing_ChooseSureThing | | | | | | | |
| Putamen (49) | Left | 231 | -24 | 8 | 6 | 5.03 | <.001 |
| Putamen (49) | Right | 336 | 24 | 8 | -4 | 4.68 | <.001 |
| Temporal/Parietal Lobe (37) | Right | 325 | 54 | -52 | 10 | 4.65 | <.001 |
| Globus Pallidus | Left | 152 | -20 | -12 | -4 | 4.16 | =.005 |
| Supramarginal Gyrus (40) | Left | 159 | -52 | -26 | 34 | 4.03 | =.004 |
| Frontal Lobe (10) | Left | 136 | -24 | 36 | 16 | 4.0 | =.009 |
| SureThing-GamblePenalty+GambleReward_ChooseSureThing | | | | | | | |
| Inferior Occipital Lobe (19) | Right | 204 | 38 | -82 | -10 | 4.49 | =.001 |
| Parietal Lobe (39) | Right | 133 | 36 | -62 | 46 | 3.59 | =.011 |

* = Small volume corrected using WFU PickAtlas Utilizing a mask of brodmann’s area 24 and 32 with 0 < Y < 36 and Z > 5, with a dilation of 3mm.

| Region  (Closest Brodmann Area) | Laterality | Cluster Size | Peak X | Peak Y | Peak Z | Max stat Z | P Cluster Corrected |
| --- | --- | --- | --- | --- | --- | --- | --- |
| GamblePenalty-Saccade_ChooseGamble | | | | | | | |
| Inferior Occipital Lobe (18) | Right | 209 | 38 | -90 | -8 | 4.44 | <.001 |
| Inferior Occipital Lobe (18) | Left | 125 | -36 | -88 | -14 | 4.06 | =.004 |
| Cingulate Gyrus (8) | Bilateral | 63† | 4 | 16 | 44 | 4.00 | =.002* |
| Parietal Lobe (7) | Right | 87 | 22 | -60 | 52 | 3.82 | =.029 |
| GambleReward-Saccade_ChooseGamble | | | | | | | |
| Anterior Insula (13) | Right | 105 | 34 | 26 | -2 | 4.94 | =.013 |
| Middle Frontal Gyrus (6) | Left | 194 | -34 | -10 | 50 | 4.70 | <.001 |
| Inferior Occipital Lobe (18) | Right | 250 | 30 | -90 | -6 | 4.18 | <.001 |
| Supplementary Motor Area (6) | Bilateral | 313 | -4 | 6 | 56 | 4.11 | <.001 |
| Occipital Lobe (18) | Left | 125 | -34 | -88 | -10 | 3.69 | =.005 |
| SureThing-Saccade_ChooseGamble | | | | | | | |
| Cingulate Gyrus (32/8) | Bilateral | 218 | 6 | 24 | 40 | 4.73 | <.001 |
| Parietal Lobe (40) | Right | 211 | 46 | -42 | 50 | 4.19 | <.001 |
| GambleReward+GamblePenalty-Saccade_ChooseGamble | | | | | | | |
| Inferior Occipital Lobe | Right | 436 | 34 | -84 | -8 | 4.94 | <.001 |
| Superior Frontal Gyrus |  | 958 | -14 | 6 | 62 | 4.71 | <.001 |
| Superior Parietal Lobe | Right | 547 | 26 | -52 | 42 | 4.69 | <.001 |
| Angular Gyrus | Left | 357 | -32 | -52 | 36 | 4.61 | <.001 |
| Angular Gyrus | Right | 114 | 50 | -52 | 36 | 4.48 | =.001 |
| ﻿Inferior Occipital Lobe (37) | Left | 170 | -40 | -52 | -20 | 4.47 | <.001 |
| Inferior Occipital Lobe (19) | Right | 377 | -34 | -86 | -12 | 4.41 | <.001 |
| GambleReward-GamblePenalty_ChooseGamble & GamblePenalty-GambleReward_ChooseGamble | | | | | | | |
| none | none | none | none | none | none | none | none |
| GambleReward+GamblePenalty-SureThing_ChooseGamble | | | | | | | |
| none | none | none | none | none | none | none | none |
| SureThing-GambleReward+GamblePenalty_ChooseGamble | | | | | | | |
| none | none | none | none | none | none | none | none |

**Binary Logistic Results (within trial):**

Probability of choosing the gamble based upon fixation duration:

**(GambleReward - GamblePenalty)/(GambleReward +** **GamblePenalty):** (*t*(17 = 3.91, *p* = .001)

**ST - (GambleReward + GamblePenalty) / ST + (GambleReward + GamblePenalty):** (*t*(17 = -5.43, *p* < .001).

B Values per subject:

| Subject | WinLoss B | GambleST B | |
| --- | --- | --- | --- |
| 201 | 1.87 | -1.2 |  |
| 202 | -0.848 | -1.38 |  |
| 203 | 2.73 | -10.31 |  |
| 204 | 0.911 | -0.07 |  |
| 205 | 2.69 | -3.18 |  |
| 207 | 0.25 | -0.81 |  |
| 208 | -0.96 | -2.35 |  |
| 209 | 0.51 | -2.32 |  |
| 210 | 0.15 | -1.71 |  |
| 211 | 3.78 | -2.68 |  |
| 213 | 2.5 | -4.77 |  |
| 215 | 0.8 | -5.87 |  |
| 218 | 1.17 | -4.03 |  |
| 217 | 0.255 | -0.76 |  |
| 220 | 0.35 | -4.39 |  |
| 221 | 4.63 | -6.27 |  |
| 222 | 2.24 | -1.71 |  |
| 223 | 2.56 | -6.17 |  |

**Linear Regression (across trial)**

Across trials, only total time spent fixating on combined possible gamble outcomes relative to the sure-win reward predicted a greater percentage of gambles chosen (*t*(17 = 5.65, *p*=.030).

**Percent of Gambles Chosen Per Subject**

| ID | % Gamble Choice |
| --- | --- |
| 201 | 29.09090909 |
| 202 | 38.18181818 |
| 203 | 52.77777778 |
| 204 | 32.72727273 |
| 205 | 14.28571429 |
| 207 | 10.71428571 |
| 208 | 35.8490566 |
| 209 | 50.90909091 |
| 210 | 29.09090909 |
| 211 | 14.54545455 |
| 213 | 43.63636364 |
| 215 | 37.5 |
| 218 | 18.18181818 |
| 217 | 55.35714286 |
| 220 | 32.72727273 |
| 221 | 27.45098039 |
| 222 | 57.14285714 |
| 223 | 62.74509804 |

**Tertiary Imaging Analyses**

**Option Values Within Trials During Choice**

Two parametric analyses were undertaken to determine whether neural activation during choice was associated with the relative values of the ST and Gamble options. Neural activation was investigated during choice across all trials according to the value of the G-Reward relative to G-Penalty (GambleReward - GamblePenalty)/(GambleReward+GamblePenalty) and the SureThing relative to the Gamble (ST - (GambleReward + GamblePenalty)) / (ST + (GambleReward + GamblePenalty)). No significant clusters emerged from these analyses at the level of whole-brain or small volume correction (X=0, Y=36,Z=5, with a dilation of 3mm).

**Option Values Across Trials During Foveations**

Three parametric analyses were undertaken to determine whether neural activation across trials during foveations on the GambleReward, GamblePenalty, and SureThing options was associated with their values. No significant clusters emerged from these analyses at the level of whole-brain or small volume correction (X=0, Y=36,Z=5, with a dilation of 3mm).. It is noted that the current study was not designed with these analyses in mind and that the ranges of values for the GambleReward, GamblePenalty, and SureThing options across the seven different gambles are very wide, without even sampling of values between the numbers (e.g. GambleReward only included seven different values with a minimum of 10 and a maximum of 5000, without equal spread (**Table S1)**).

**Links to De-identified Data:**

**De-faced Anatomical Scans (.nii.gz):**<https://figshare.com/s/42186016ccb312aa5e6e>

**Raw Functional Scans (.nii.gz):** <https://figshare.com/s/a57da73bc727e5287e3d>

**Figure Data (.nii.gz):** <https://figshare.com/s/8aa9b28a22b38fa528e2>

**Raw Eye-tracking Data:** <https://figshare.com/s/8c5a1da369ee651ee9fc>

**Behavioral Preproc Data:**ttps://[figshare.com/s/50c175b49247124561f8](http://figshare.com/s/50c175b49247124561f8)

**Behavioral Matlab Input Data:**<https://figshare.com/s/7821e35b776ba70eb4bb>

**Imaging Processing Scripts:**<https://figshare.com/s/e48ee2d34f8f61dad658>
